# Relationship between CRP and hypofibrinolysis: Is this a possible mechanism to explain the association between CRP and outcome in critically ill patients?

**DOI:** 10.1186/1477-9560-2-7

**Published:** 2004-09-30

**Authors:** Karim Zouaoui Boudjeltia, Michael Piagnerelli, Dany Brohée, Michel Guillaume, Philippe Cauchie, Jean-Louis Vincent, Claude Remacle, Yves Bouckaert, Michel Vanhaeverbeek

**Affiliations:** 1 Experimental Medicine Laboratory, ULB 222 Unit, ISPPC, CHU A. Vésale, Montigny-le-Tilleul, Belgium; 2 Dept of Intensive Care Medicine, Erasme Hospital, Free University of Brussels, Belgium; 3 Dept of Cardiology, ISPPC, CHU A.Vésale, Montigny-Le-Tilleul, Belgium; 4 Institute of life sciences, Laboratory of cellular Biology, Université Catholique de Louvain, Louvain-La-Neuve, Belgium; 5 Dept of Intensive Care Medicine, Tivoli Hospital, La Louvière, Belgium

**Keywords:** C-reactive protein, acute phase reactant, euglobulin clot lysis time, inflammation, sepsis, endothelium dysfunction.

## Abstract

**Background-:**

Endothelial cell dysfunction may be implicated in the development of multiple organ failure (MOF) by a number of mechanisms. Among these, altered fibrinolysis promotes fibrin deposition, which may create microvascular alterations during inflammation. Elevated concentrations of C-reactive protein (CRP), especially when these persist over time, are correlated with an increased risk of MOF and death. CRP may inhibit fibrinolysis by inducing plasminogen activator inhibitor-1 (PAI-1) release from human aortic endothelial cells. Moreover, the administration of recombinant CRP in volunteers may increase circulating PAI-1 levels.

In this study, we tested the hypothesis that CRP is associated with hypofibrinolysis in intensive care patients with and without sepsis.

**Methods-:**

We studied the association of inflammation and abnormal fibrinolysis in intensive care unit (ICU) patients with (n = 11) and without (n = 21) sepsis. The inflammatory response was assessed by serum concentration of C-reactive protein (CRP), a marker of the acute phase reaction, which increase rapidly in the inflammatory response, and the plasma fibrinolytic capacity was evaluated by the Euglobulin Clot Lysis Time (ECLT), determined by a new semi-automatic method.

**Results-:**

ECLT was significantly higher in septic than non-septic patients (1104 ± 439 vs 665 ± 275 min; p = 0.002) and was significantly correlated with CRP concentration (R^2 ^= 0.45; p < 0.001). In a multivariate analysis, CRP was the strongest predictor of ECLT (R^2 ^= 0.51, F = 25.6, p < 0.001). In addition, the overall ICU length of stay was significantly correlated with CRP (R^2 ^= 0.264, p = 0.003) and ECLT (R^2 ^= 0.259, p = 0.003).

**Conclusion-:**

In critically ill patients a significant correlation thus exists between plasma fibrinolytic capacity and serum CRP levels. Our data were obtained in the first 24 hours of ICU admission or of sepsis, thus, the relation between CRP and hypofibrinolysis appeared very quickly. This finding is compatible with a link between inflammation and abnormal fibrinolysis, and may explain the negative prognostic value of CRP in critically ill patients.

## Background

Endothelial cells have a key role in the control of vascular permeability and vessel tone, coagulation and fibrinolysis, and inflammatory response [1]. There is an increasing body of evidence supporting the critical role of the vascular endothelium in the pathogenesis of multiple organ failure (MOF) in critically ill patients [2].

Endothelial dysfunction/or activation is associated with an imbalance in hemostatic functions. Endothelial cells are responsible for the release of tissue plasminogen activator (t-PA) and contribute to the release of plasminogen activator inhibitor-1 (PAI-1). Inhibition of the fibrinolytic system amplifies the pathogenic role of fibrin deposition during severe inflammation [3]. Multiple factors, including lipoproteins, cytokines, and inflammatory proteins can modulate the endothelial cells to produce t-PA and PAI-1 [4].

In infected patients, elevated concentrations of serum CRP are correlated with a risk of MOF and death [5], especially when these persist over time [5]. However, the possible biological involvement of CRP in the development of MOF and death is unknown.

CRP can act directly on endothelial cells, inducing, for example, the expression of intercellular adhesion molecule (ICAM)-1 [6] and the production of inflammatory cytokines such as interleukin- (IL)-6 [7]. It may also inhibit fibrinolysis by inducing PAI-1 release from human aortic endothelial cells [8]. Moreover, the administration of recombinant CRP in volunteers may increase circulating PAI-1 levels [9].

In this study, we tested the hypothesis that CRP is associated with hypofibrinolysis as measured by ECLT in intensive care patients with and without sepsis.

## Material and methods

### Subjects

After approval by the A. Vésale hospital ethics committee, we studied 32 ICU patients with severe sepsis (n = 11) or other diagnoses (n = 21). Infection definition required isolation of a microorganism from a normally sterile body site, concurrent with accompanying signs and symptoms of sepsis and decision of antibiotic therapy. Criteria for severe sepsis included signs of at least one organ dysfunction attributed to sepsis [10]. All patients (septic and non-septic) were enrolled in the first day of ICU admission to limit the delay of inflammatory response. The exclusion criteria were: antibiotics treatment in the non-septic group except for surgical prophylaxis, red blood cell transfusion in the last 72 h, active hemorrhage, hematological disorders, recent cytotoxic chemotherapy, burns, cardiogenic shock, cirrhosis, pregnancy. The simplified acute physiologic score (SAPS II score) [11] was determined in each patient during the first 24 hours after admission.

### Blood samples

Blood samples were obtained during the first 24 hours of sepsis or on the first day of admission for non-septic patients. Serum samples were collected in vacuum tubes without anticoagulant. Plasma samples were harvested in citrated vacuum tubes and put in melting ice. Whole blood was collected on EDTA-treated tubes. CRP was evaluated by antibody-binding and turbidity measurement on SYNCHRON LX^®^. Fibrinogen was determined by thrombin time on a STA^® ^automate (STAGO). Leukocyte and platelet counts were determined on a hemocytometer (CELL-DYN4000^®^, ABBOTT). All tests were performed on blood obtained from the same venipuncture.

### Plasma fibrinolytic capacity

The Euglobulin Clot Lysis Time (ECLT), which is the most common test used to estimate the plasma fibrinolytic capacity, represents the balance between t-PA and PAI-1 activities [12]. ECLT was measured on fresh plasma the same day as other parameters by a method described elsewhere [13]. Briefly, we designed a completely computerized, semi-automatic, 8-channel device for measurement and determination of fibrin clot lysis (Lysis Timer, EREM, Belgium). The lysis time is evaluated by a mathematical analysis of the lysis curve and the results are expressed in minutes (range: 5 to 9999). The efficiency scores of the method are <4% in intra-assay and <7% in inter-assay.

### Statistics

We used SigmaStat^® ^software package (Jandle Scientific). The data are presented as mean ± SD. Correlation between variables was analyzed using a Pearson correlation test. A multivariate analysis was used with stepwise backward selection of the explicative variables. Sepsis was considered as a dichotomous variable while all other data were considered as continuous (CRP, fibrinogen, leukocyte, monocyte and platelet counts). ECLT was the dependent variable. A probability level of p < 0.05 was considered as statistically significant.

## Results

The major cause of severe sepsis (9 patients) or septic shock (2 patients) was pneumonia (8 patients); angiocholitis was the cause in 1 patient, and in 2 patients the cause was not identified. In 8 patients (7 with pneumonia and 1 with angiocholitis), the infection was due to a Gram negative bacteria. Only 1 patient had documented bacteremia. Non-septic patients were admitted for postoperative surveillance (7 patients), intracerebral hemorrhage (3 patients), heart failure (3 patients), drug intoxication (3 patients), or aggravated chronic obstructive pulmonary disease (5 patients).

**Figure 1:**
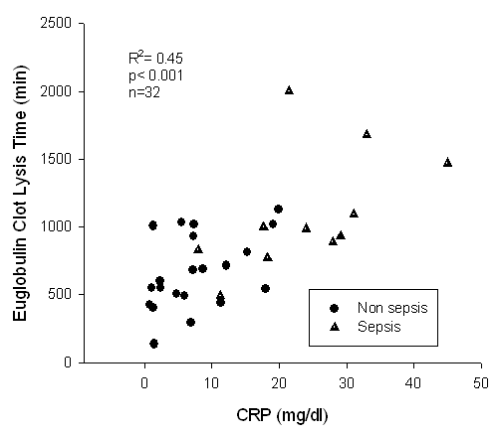
Correlation between serum CRP levels and Euglobulin Clot Lysis Time.

As expected, inflammatory parameters such as white blood cells and CRP levels, the SAPS II score and ECLT were higher in the septic than the non-septic population (Table 1). In an univariate analysis (in all patients), the ECLT was strongly correlated with serum CRP concentrations (R^2 ^= 0.45; p < 0.001) with no perceptible threshold (Fig 1). Surprisingly, there was no relationship between the SAPS score and ECLT (R^2 ^= 0.08; p = 0.15). In multivariate analysis, ECLT was best predicted by the CRP level (R^2 ^= 0.51; F = 25.6; p < 0.001) and not significantly by sepsis or the fibrinogen concentration. Interestingly, the ICU length of stay was significantly correlated with CRP (R^2 ^= 0.264, p = 0.003) and ECLT (R^2 ^= 0.259, p = 0.003) in all patients, and in the survivors (R^2 ^= 0.13, p = 0.05 and R^2 ^= 0.3, p = 0.003, respectively).

**Table 1:** Population characteristics

	Sepsis (n = 11)	Non-sepsis (n = 21)	p value
Age, years	68 ± 19	67 ± 17	0.95
SAPS II	47 ± 11	25 ± 14	0.001
ICU stay, days	11.6 ± 7.8	6.7 ± 6.8	0.07
Death, n (%)	2 (18)	3 (14)	0.57
Leukocytes (×10^3 ^cells/μl)	10.4 ± 4.5	10.9 ± 3.7	0.74
Monocytes (×10^3 ^cells/μl)	549 ± 225	711 ± 398	0.24
Platelets (×10^3 ^cells/μl)	207 ± 157	234 ± 94	0.54
Fibrinogen (mg/dl)	657 ± 123	445 ± 132	<0.001
CRP (mg/dl)	24.2 ± 10.5	7.6 ± 6.1	<0.001
ECLT (min)	1104 ± 439	665 ± 275	0.002

Mean ± SD; p value (t-stest).

SAPS : Simplified Acute Physiologic Score; ICU : Intensive Care Unit; CRP : C-Reactive Protein;

ECLT : Euglobulin Clot Lysis Test

## Discussion

Many studies have demonstrated that sepsis is associated with endothelial cell dysfunction and promotes coagulation activation [14]. Although levels of t-PA antigen increase in sepsis, fibrinolysis is inhibited by increased levels of PAI-1. ECLT is, therefore, an interesting test, because it represents the balance between t-PA and PAI-1 activities. Previously considered as an imprecise method, we have been able to improve the precision and reproducibility of the test with a new semi-automatic device [13].

Inhibition of the fibrinolytic system may contribute to MOF, as fibrin can activate endothelial cells leading to a disorganization of the monolayer and the release of inflammatory cytokines [4]. Increased CRP levels are associated with worse outcomes and MOF in ICU patients [3]. The role of CRP on fibrinolysis is unknown *in vivo*. Our data suggest that CRP could itself be involved in the processes leading to endothelium dysfunction. The observed relationship does not prove a direct biological link between increasing CRP and hypofibrinolysis; however, indirect arguments exist in support of the concept.

Several *in vitro *studies have reported the direct effects of CRP on endothelial cells [6-8]. *In vivo*, Cleland et al. [15] reported a relationship between serum CRP levels and the forearm blood flow response to N^G^-monomethyl-L-arginine (L-NMMA), reflecting endothelial dysfunction. Bisoendial et al. reported that the administration of CRP in volunteers impairs the fibrinolytic balance [9]. In addition, CRP has a strong prognostic value in acute coronary syndromes [16]. In a non-selected population with no inflammatory syndrome (CRP below 1.5 mg/dl, n = 160), we also observed that ECLT was significantly correlated with serum CRP levels [17].

CRP could also act indirectly on endothelial cells via the action of monocytes and the release of tumor necrosis factor-α (TNF-α). TNF-α is a strong inducer of PAI-1 production *in-vitro *and *in-vivo*. This mechanism seems to be important in sepsis, as high plasma levels of PAI-1 are associated with poor outcome [18]. Moreover, an association between CRP and TNF-α has also been described [19]. CRP can induce the monocyte release of cytokines such as IL-1β, IL-6, and also TNF-α through Fc receptors (γRI/CD64, FcγRIIa/CD32) [20].

CRP also has essential biological functions. No polymorphism of either the gene coding sequence or of the protein itself has been described in humans [21]. Also, high levels of human CRP protect against lethal infection. Transgenic mice capable of produce human CRP are protected against lethal infection by Gram positive and negative bacteria, Szalai et al [22,23].

This work is a pilot study. We have chosen to include patients in the first day of ICU admission to limit the possible rapid effect of inflammatory response on fibrinolysis, especially in septic patients. Indeed, this particular patient population has inflammatory reaction before signs of severe sepsis and thus before their admission to ICU. In fact, we have studied ECLT test at the onset of the organ dysfunction and the inflammatory reaction. Other studies with serial measurement of ECLT in patients who developed nosocomial ICU infections are needed to study the time course of these events. Moreover, we could not definitively exclude that all patients in the non-septic groups were non infected. For example, some non-septic patients with decompensated COPD may have minor infections, despite the negative microbiology cultures and the absence of antibiotic therapy. Viral infections were also possible in some patients.

Moreover, it would be of great interest to determine the interactions between CRP and ECLT with IL-6, TNF-α and endothelium dysfunction markers such as soluble thrombomodulin and soluble von Willebrand factor.

## Conclusion

Despite accumulating evidence that the inflammatory and coagulation systems are activated in sepsis, little is known about the mechanisms that ultimately lead to organ dysfunction and death. Our data were obtained in the first 24 hours of ICU admission or of sepsis, thus, the relation between CRP and hypofibrinolysis appeared very quickly. Prospective studies including the time course of CRP and hypofibrinolysis would provide additional information about this relationship.

## Authors' contributions

KZB: Laboratory analysis, writing of the manuscript and design of the study.

MP: patients recruitment and design of the study.

DB: coordination and design analysis of the results.

MG: design of the study.

PC: laboratory analysis.

JLV: design of the study and analysis of the results.

CR: design of the study and analysis of the results.

YB: patients recruitment.

MV: statistical analysis and coordination.
